# 肺肉瘤样癌的诊治现状

**DOI:** 10.3779/j.issn.1009-3419.2018.12.07

**Published:** 2018-12-20

**Authors:** 雷 刘, 若川 臧, 朋 宋, 树庚 高

**Affiliations:** 100021 北京，国家癌症中心/国家肿瘤临床医学研究中心/中国医学科学院北京协和医学院肿瘤医院胸外科 Department of Thoracic Surgery, Nationanl Cancer Center/National Clinical Research Center for Cancer/Cancer Hospital, Chinese Academy of Medical Scienses and Peking Union Medical College, Beijing 100021, China

**Keywords:** 肺肿瘤, 肺肉瘤样癌, 诊断, 治疗, 预后, Lung neoplasms, Pulmonary sarcomatoid carcinoma, Diagnosis, Treatment, Prognosis

## Abstract

肺肉瘤样癌（pulmonary sarcomatoid carcinoma, PSC）是一类罕见的非小细胞肺癌的统称，约占肺恶性肿瘤的0.1%-0.5%，按照2015年世界卫生组织（World Health Organization, WHO）对于肺恶性肿瘤分类标准，属于肺恶性上皮细胞肿瘤，包含5个亚型：多形性癌、梭形细胞癌、巨细胞癌、癌肉瘤和肺母细胞瘤。该类疾病无典型临床症状，但影像学有一定特点，确诊需要病理和免疫组化检查。治疗原则与其他非小细胞肺癌相似，早期患者是以手术为主的综合治疗模式。但对放化疗不敏感，容易复发和转移，预后不良。随着分子病理学发展，靶向治疗和免疫治疗可能会有广阔的应用前景。

## Abstract (en)

Pulmonary sarcomatoid carcinoma (PSC) is a rare, poorly differentiated, subtype of non-small cell lung carcinoma (NSCLC) and constitutes approximately 0.1% to 0.5% of all lung malignancies. PSC can be divided into five subtypes based on the 2015 World Health Organization (WHO) classification of lung tumors: pleomorphic carcinoma, spindle cell carcinoma, giant cell carcinoma, carcinosarcoma, and pulmonary blastoma. Some imaging characteristics can be found for PSC although no special symptoms. The accurate pathological diagnosis of PSC can be a significant challenge, which depends on pathology and immunohistochemistry. PSC should be managed similar to other NSCLC, surgical resection is the standard management for early stage cases, moreover, multimodal treatment should be considered. However, PSC is insensitive to radiotherapy and chemotherapy, and has high rate of local and metastatic recurrence and poor prognosis. With the development of molecular pathology, targeted therapy and immunotherapy may have broad prospects.

肉瘤样癌（sarcomatoid carcinoma, SC）是一类少见的恶性肿瘤，兼有上皮和间叶肿瘤的特征，可以发生在多种器官，比如皮肤、骨骼、甲状腺、乳腺和肺等^[1]^。肺肉瘤样癌（pulmonary sarcomatoid carcinoma, PSC）罕见，占肺恶性肿瘤的0.1%-0.5%^[2, 3]^，报道较少，迄今为止，临床诊治方案、预后和影响因素都存在争议，现综述如下。

## 1 病理特征

PSC是一类罕见的肺恶性肿瘤的总称，属于非小细胞肺癌（non-small cell lung cancer, NSCLC）范畴。此类肿瘤在1981年被称为“鳞状细胞癌的一种变体（a variant of squamous cell carcinoma）”；1999年，被更名为“癌含有多形细胞、肉瘤样或肉瘤成分（carcinomas with pleomorphic, sarcomatoid or sarcomatous elements）”；2004年，世界卫生组织（World Health Organization, WHO）根据肺肿瘤病理形态学特征，规定其归属于肺恶性上皮细胞肿瘤，有了一个统一的名称，即“肺肉瘤样癌”^[4]^，在2015版WHO肺肿瘤分类中，PSC的名称、诊断标准变化不大，共有5个亚型，分别为：多形性癌、梭形细胞癌、巨细胞癌、癌肉瘤和肺母细胞瘤^[5]^。目前组织学研究认为，PSC是一组起源于相同原始上皮、经上皮-间质转化（epithelial-mesenchymal transition, EMT）后形成的一组转化性癌^[6, 7]^。

大体形态^[8]^：可为中央型或外周型，多位于上叶。癌肉瘤主要为中心型，多形细胞癌和肺母细胞瘤多为外周型。肿瘤多体积较大，且侵犯胸壁。大体形态上边界较清晰，伴有坏死和出血，质地可软，也可坚硬，或呈胶样。单纯从大体形态上，难以与其他类别NSCLC相鉴别。

组织学形态^[5]^：①多形性癌：最多见的亚型，指鳞状细胞癌或腺癌或未分化的NSCLC中至少含有10%的梭形细胞和（或）巨细胞，或只含有梭形细胞或巨细胞的分化差的NSCLC；②梭形细胞癌：几乎全部为梭形细胞构成；③巨细胞癌：几乎全部为巨细胞（包括多核巨细胞）构成；④癌肉瘤：NSCLC（主要为鳞癌和腺癌）和伴有异源性成分的肉瘤（如横纹肌肉瘤、软骨肉瘤、骨肉瘤）构成的恶性混合型肿瘤；⑤肺母细胞瘤：由原始上皮成分和间叶成分构成。

## 2 临床特征

PSC好发于老年男性，重度吸烟患者多见，男女比例约1.5:1^[9]^，平均年龄65岁-75岁^[10]^。常见表现为胸痛、咳嗽、咳血、呼吸困难和体质量下降等^[1]^，大约70%患者就诊时为局部晚期或远处转移^[11]^。PSC复发、远处转移常见，常见的转移部位是肺、骨、肾上腺、胸膜和脑^[11]^，还有皮肤转移瘤、小肠转移瘤^[12, 13]^，90%的肺肉瘤样癌存在血管侵犯，其容易复发转移可能与此有关^[14]^。

PSC在计算机断层扫描（computed tomography, CT）上的表现有一定特点：肿瘤多为单发；直径较大，多 > 3 cm，有的可达到18 cm；中心型或外周型，圆形，密度均匀，边缘光滑，可有毛刺或分叶，钙化少见；增强CT呈环形或斑片状强化^[10, 15, 16]^；胸壁侵犯和（或）胸腔积液征象^[11]^。另有学者在正电子发射计算机断层显像（positron emission tomography-CT, PET-CT）检查中发现，和普通NSCLC相比，PSC有更高的平均SUVmax值（PSC组*vs*普通NSCLC组，15.11 *vs* 7.66）^[17]^。

## 3 诊断

PSC的临床特征和影像学检查虽然有一定特点但确诊仍需要病理和免疫组化检查^[15]^。该类肿瘤肿瘤-淋巴结-转移（tumor-node-matastasis, TNM）分期和肿瘤分级同其他NSCLC。

PSC各类的组织形态学特点前面已述，但在组织细胞形态学上难以确定时，免疫组化检查就起着非常重要的作用，可以用来鉴别诊断^[18]^。目前临床上针对肺肉瘤样癌常用的免疫组化检测指标主要包括两大类：一类是上皮生物学标记物，包括：细胞角蛋白（cytokeratin, CK）、上皮细胞膜抗原（epithelial membrane antigen, EMA）、抗细胞角蛋白单克隆抗体（anti-pan cytokeratin antibody, AE1/AE3）、甲状腺转录因子1（thyroid transcription factor 1, TTF-1）、癌胚抗原（carcinoembryonic antigen, CEA）、CAM5.2、细胞角蛋白7（cytokeratin 7, CK7）、p40、细胞角蛋白5/6（cytokeratin 5/6, CK5/6）等；另一类是间质细胞生物学标记物，包括：波形蛋白（vimentin）、结蛋白（desmin）等^[4, 19]^。在肺肉瘤样癌上皮和肉瘤两种成分区域，上皮标记物和间质标志物均可呈阳性表达^[19]^。另外，PSC和恶性黑色素瘤、间皮瘤、肉瘤等在免疫组化上有重叠之处，需要鉴别^[18]^。

获取组织学标本常用的方法有手术、穿刺活检、支气管镜活检等。PSC术前确诊非常困难，而且部分晚期患者有可能会因为无法获得组织标本或组织标本量少而漏诊或被诊断为其他类型的NSCLC。Lin等^[20]^报道69例PSC经支气管镜活检或经皮肺穿刺活检，只有8例在术前确诊（11.6%），其他被误诊为鳞癌或腺癌，误诊的原因包括肿瘤的多分化趋势、组织量少和不典型的细胞形态，而且大多数术中冰冻病理结果和最终病理结果也不一致。2015版WHO肺肿瘤病理分类中指出，多形细胞癌、梭形细胞癌、巨细胞癌3种不能通过小活检和细胞学来诊断，癌肉瘤和肺母细胞瘤在小活检和细胞学上诊断也非常困难^[5]^。因此，需要有足够的外科标本，结合免疫组化和显微镜检查才能获得明确诊断^[8]^。但临床上PSC多数为晚期患者，获得足够的外科标本常常有困难，所以如何从小标本获得诊断是临床的一个亟待解决的问题，Pelosi等^[6]^提出了改良的波形蛋白组织学评分（modified vimentin histologic score, M-VHS），对活检标本和外科标本进行对比，发现评分大致相当，所以提出M-VHS对小活检标本的诊断有一定价值。

目前发现在PSC肿瘤组织中存在多个基因异常，有报道PSC的肿瘤突变负荷（tumor mutational burden, TMB）明显高于非PSC的NSCLC（20% *vs* 14%, *P*=0.056）^[21]^。最常见的有*EGFR*、*TP53*、*KRAS*、*ALK*和*MET*等，这些基因改变可单独存在，也可同时发生^[19]^。*EGFR*、*TP53*、*KRAS*、*ALK*和*MET*的突变率分别为0%-28.1%、58%-74%、3%-34%、3.5%-10.7%和13.6%-31.8%^[21-26]^，另外还有*PIK3CA*、*STK11*突变，*ALK*易位，*CDK4*、*BRAF*、*HER2*、*RET*等基因组改变^[21, 27]^。其中*EGFR*、*ALK*和*MET*均为PSC重要的驱动基因，可以作为靶向治疗的参考依据。*KRAS*突变和不良预后显著相关，其检测对判断PSC的预后具有参考价值^[28]^。另外，Lococo等^[29]^发现PD-L1表达与总的基因突变负荷呈正相关，尤其是*KRAS*突变状态相关（PD-L1阳性率在*KRAS*突变型和野生型分别为44.4%和12.0%）。由于PSC中基因异常比较常见，多数学者推荐对其进行更多基因的检测，进行深入研究，也许能给部分患者带来新的治疗机会^[21]^。

## 4 治疗

治疗原则同其他NSCLC^[30]^，是以手术为主的综合治疗策略。对于可切除肿瘤，应行肺叶切除或全肺切除加纵隔淋巴结清扫术，化疗（以铂类为基础的联合化疗方案）和放疗也可选择，靶向治疗和免疫治疗有应用前景。

对于早期PSC，手术仍然是首选治疗方案^[11, 20]^。以铂类为基础的联合化疗方案是NSCLC的标准辅助化疗和姑息化疗方案，也通常用于PSC。但由于PSC发病率低，对照研究少，所以化疗对PSC的治疗价值尚无定论^[10, 20]^，各类报道结论差异较大。Karim等^[2]^回顾性对比了手术加化疗、单纯手术、单纯化疗和单纯观察的治疗效果，中位生存期分别为457.6 d（95%CI: 206-1, 187）、713.5 d（95%CI: 246-1, 138）、256 d（95%CI: 114-600）和205.5 d（95%CI: 98-447），认为进展期PSC患者似乎未能从化疗中获益，但作为术后辅助治疗手段还是可以考虑。Lin等^[20]^研究认为对早期PSC患者，新辅助化疗和辅助化疗均不能带来生存获益；对于晚期PSC，应用吉西他滨联合顺铂（GP）或紫杉醇联合顺铂（TP）方案化疗，客观缓解率达21.9%，中位生存时间14个月，疗效和同期NSCLC近似^[31]^。另一项多中心研究纳入97例晚期PSC患者，应用含或不含铂类的一线化疗方案，中位生存期6.3个月^[32]^。化疗对晚期PSC疗效的差异，可能与各研究中术后复发转移患者和初始为Ⅳ期患者的比例不同有关，需要进一步研究。总体来说，PSC对多种化疗药的耐药可能是其化疗效果差、预后差的原因^[10]^。

2015版WHO肺癌分类中，建议含有腺癌成分的肉瘤样癌行*EGFR*突变和*ALK*基因融合检测^[5]^，可以给一部分晚期PSC患者带来新的治疗机会。在PSC中*EGFR*突变率不一，有报道22例患者中无*EGFR*突变^[33]^；另有33例PSC患者中统计，有9例*EGFR*突变^[24]^。Chen报道141例PSC患者中，ALK重排阳性者5例，占3.5%，同其他类型NSCLC的阳性率相似^[22]^。但TKI在PSC中应用的报道很少。有报道1例肺多形细胞癌术后复发患者，*EGFR*外显子19缺失，接受吉非替尼治疗，获得了35个月完全缓解^[34]^。牛海涛等^[26]^报道84例PSC患者中，*ALK*融合基因阳性者9例（10.7%），均接受克唑替尼治疗，1例疗效评估为完全缓解，6例部分缓解，2例疾病稳定，其1年、3年、5年生存率分别为100%（9/9）、100%（9/9）和88.9%（8/9）。但也有学者认为单独TKI治疗可能无效^[24]^。*c-MET*被认为是继*EGFR*基因突变和*ALK*基因融合之后，NSCLC又一个重要驱动基因，是NSCLC潜在的治疗靶点^[25]^。Liu等^[35]^报道*MET*基因突变导致外显子14的转录后缺失在PSC中常见，突变率为22.2%（8/36），克唑替尼能有效抑制*MET*外显子14突变细胞株的生长，同时报道1例*MET*外显子14突变患者服用克唑替尼后疗效显著。另外*BRAF*突变也较常见，近期有报道1例*BRAF* V600E突变的晚期PSC患者，接受威罗非尼治疗10个月，疗效评价部分缓解（partial response, PR）^[21]^。Lococo等^[29]^分析了43例外科手术后的PSC，发现25%的PSC患者PD-L1显著表达，Kim等^[36]^报道41例肺多形细胞癌中PD-L1和PD-L2表达率分别为90.2%和87.8%。但PD-L1抑制剂在PSC中的应用多为个案报道，Schrock等^[21]^报道1例Ⅳ期PSC患者应用派姆单抗10个月，疗效评价PR，并指出肿瘤突变负荷（tumor mutation burden, TMB）高的患者对免疫治疗的应答较好，另有1例PSC伴小肠转移瘤的患者接受纳姆单抗治疗3个月后疗效评价PR，因肺炎停药，7个月后复查，病情未进展^[14]^。

## 5 预后

PSC作为一类特殊类型的NSCLC，恶性程度较高，容易复发和转移，预后不良。影响其预后的因素包括是否发生远处转移、肿瘤大小、是否完整切除、TNM分期、*ALK*融合基因状态、组织亚型等^[20, 37-39]^。

手术切除的PSC的5年生存率为12.6%-54.3%，有报道近半数（43/99）患者会出现复发或转移，平均术后复发时间为6.8个月^[39-41]^。PSC和普通NSCLC相比，是否恶性程度更高、预后更差有一些争议^[39]^，曾有研究^[42]^表明PSC和普通NSCLC预后无显著差异，但近期的报道^[39]^多数认为PSC比普通NSCLC侵袭性更高，预后更差。一项研究^[9]^分析了87, 880例手术治疗后的NSCLC生存情况，通过倾向性评分筛选，对比了1, 921例PSC和758例其他类型NSCLC生存情况，前者OS明显低于后者（HR=1.60; 95%CI: 1.31-1.97）。Martin等^[43]^报道63例接受根治性手术的PSC患者生存资料，应用1:1倾向性评分与同期1, 133例NSCLC比较，5年生存率分别为24.5%和46.3%。

综上所述，肺肉瘤样癌是一类异质性强的肿瘤的统称，发病率低，诊断有一定困难，恶性程度较高，容易复发转移，对放化疗不敏感，手术治疗是目前最有效的治疗方案。随着分子生物学的发展，可对更多的生物标志物进行检测，对PSC的诊断和治疗起到重要作用。靶向治疗和免疫治疗可能会给那些传统手段无效的患者带来新的希望。
